# Quantifying Disparities in COVID-19 Vaccination Rates by Rural and Urban Areas: Cross-Sectional Observational Study

**DOI:** 10.2196/50595

**Published:** 2024-07-19

**Authors:** Wenyong Dong, Yudong Miao, Zhanlei Shen, Wanliang Zhang, Junwen Bai, Dongfang Zhu, Ruizhe Ren, Jingbao Zhang, Jian Wu, Clifford Silver Tarimo, Theodora Ojangba, Yi Li

**Affiliations:** 1 Department of Hypertension Henan Provincial People's Hospital People's Hospital of Zhengzhou University Zhengzhou China; 2 Department of Health Management College of Public Health Zhengzhou University Zhengzhou China; 3 Department of Science and Laboratory Technology Dar Es Salaam Institute of Technology Dar es Salaam United Republic of Tanzania

**Keywords:** COVID-19 vaccination, urban and rural, the fourth COVID-19 (second booster) vaccine, disparities, China, COVID-19

## Abstract

**Background:**

Vaccination plays an important role in preventing COVID-19 infection and reducing the severity of the disease. There are usually differences in vaccination rates between urban and rural areas. Measuring these differences can aid in developing more coordinated and sustainable solutions. This information also serves as a reference for the prevention and control of emerging infectious diseases in the future.

**Objective:**

This study aims to assess the current coverage rate and influencing factors of COVID-19 (second booster) vaccination among Chinese residents, as well as the disparities between urban and rural areas in China.

**Methods:**

This cross-sectional study used a stratified random sampling approach to select representative samples from 11 communities and 10 villages in eastern (Changzhou), central (Zhengzhou), western (Xining), and northeast (Mudanjiang) Mainland China from February 1 to February 18, 2023. The questionnaires were developed by experienced epidemiologists and contained the following: sociodemographic information, health conditions, vaccine-related information, information related to the Protective Motivation Theory (PMT), and the level of trust in the health care system. Vaccination rates among the participants were evaluated based on self-reported information provided. Binary logistic regression models were performed to explore influencing factors of vaccination among urban and rural participants. Urban-rural disparities in the vaccination rate were assessed using propensity score matching (PSM).

**Results:**

A total of 5780 participants were included, with 53.04% (3066/5780) being female. The vaccination rate was 12.18% (704/5780; 95% CI 11.34-13.02) in the total sample, 13.76% (341/2478; 95% CI 12.40-15.12) among the rural participants, and 10.99% (363/3302; 95% CI 9.93-12.06) among the urban participants. For rural participants, self-reported health condition, self-efficacy, educational level, vaccine knowledge, susceptibility, benefits, and trust in the health care system were independent factors associated with vaccination (all *P*＜.05). For urban participants, chronic conditions, COVID-19 infection, subjective community level, vaccine knowledge, self-efficacy, and trust in the health care system were independent factors associated with vaccination (all *P*＜.05). PSM analysis uncovered a 3.42% difference in vaccination rates between urban and rural participants.

**Conclusions:**

The fourth COVID-19 vaccination coverage rate (second booster) among the Chinese population was extremely low, significantly lower than the previous vaccine coverage rate. Given that COVID-19 infection is still prevalent at low levels, efforts should focus on enhancing self-efficacy to expand the vaccine coverage rate among the Chinese population. For rural residents, building awareness of the vaccine’s benefits and improving their overall health status should be prioritized. In urban areas, a larger proportion of people with COVID-19 and patients with chronic illness should be vaccinated.

## Introduction

Vaccination plays an important role in preventing COVID-19 infection, reducing the severity of the disease and the case fatality rate [1-4]. Currently, the prevalence of COVID-19 infection rate is low [5]. However, COVID-19 still has a significant impact on people's health, causing issues such as long COVID-19, preterm birth, stillbirth, myocarditis, and pericarditis [6-8]. A study from Australia has shown that vaccination is effective in protecting the population [9]. Similar studies carried out in the United States indicated that COVID-19 vaccines have reduced the severity of the disease and prevented serious complications such as respiratory failure and death [10]. Other studies have shown that individuals who received booster doses of COVID-19 vaccine have stronger immune protection than those who received only a single dose [11,12]. Therefore, for countries and regions aiming to reduce the spread of COVID-19 by expanding the protection of immune barriers, increasing vaccination rates remains an important task for health care systems at this stage [13].

Establishing herd immunity through COVID-19 vaccination requires a vaccination rate ranging from 50% to 85% of the population [14,15]. An intensive vaccination campaign during the initial phase of the pandemic wave led to a lower optimal level of doses administered per 100 inhabitants (roughly 47 doses of vaccines administered) to reduce the number of infected individuals; however, as the pandemic wave progressed, the optimal level of vaccines increased to about 90 doses per 100 inhabitants to produce the same effect [16]. The maximum vaccination rate that could be achieved without a mandate was 70%, with the remaining 30% associated with people’s hesitancy toward vaccinations [17]. The factors influencing COVID-19 vaccination are complex and only partially explored. Previous studies have shown that COVID-19 vaccination was related to the severity of the outbreak, perceived susceptibility to the disease, and concerns related to the vaccine’s safety and effectiveness [18-21]. Our previous research reported correlations between COVID-19 vaccination and trust in the health care system, vaccine accessibility, lifestyle, and psychological experience [22,23]. Additionally, our previous nationwide investigation conducted during primary COVID-19 vaccination hesitancy confirmed a 2.38% gap between rural residents in China and their urban compatriots [24]. Similar findings were reported in studies related to influenza and HPV vaccines [25,26].

Disparities in COVID-19 vaccination rates between rural and urban regions are meaningful [27]. In China and other limited-income countries, frequent movement of the population between urban and rural areas is common [28]. Given the extremely high transmission capacity of COVID-2019, increasing vaccination rates among urban or rural residents unilaterally is not cost-effective in building herd immunity [29]. Currently, India, Brazil, and other limited-income countries are experiencing significant internal migration patterns similar to China’s [30,31]. Effective public governance improves prevention and preparedness to face pandemic threats [32-34]. Quantifying disparities in COVID-19 vaccination rates in rural and urban areas based on Chinese national-level evidence is crucial for global efforts to end the COVID-19 pandemic and can provide a reference for the prevention and control of emerging infectious diseases.

Therefore, we conducted a nationwide survey during the fourth COVID-19 (second booster) vaccination period. This study aims to assess the current coverage rate of COVID-19 vaccination among Chinese residents, identify influencing factors, and examine disparities between urban and rural areas in China. This study’s findings will reveal the real-time status of COVID-19 vaccination among people in China. A quantitative assessment of the disparity between urban and rural areas will deepen our understanding of the complexity of COVID-19 infection prevention, helping produce a more coordinated and sustainable solution to end the pandemic.

## Methods

### Sample and Data

**Figure 1:**
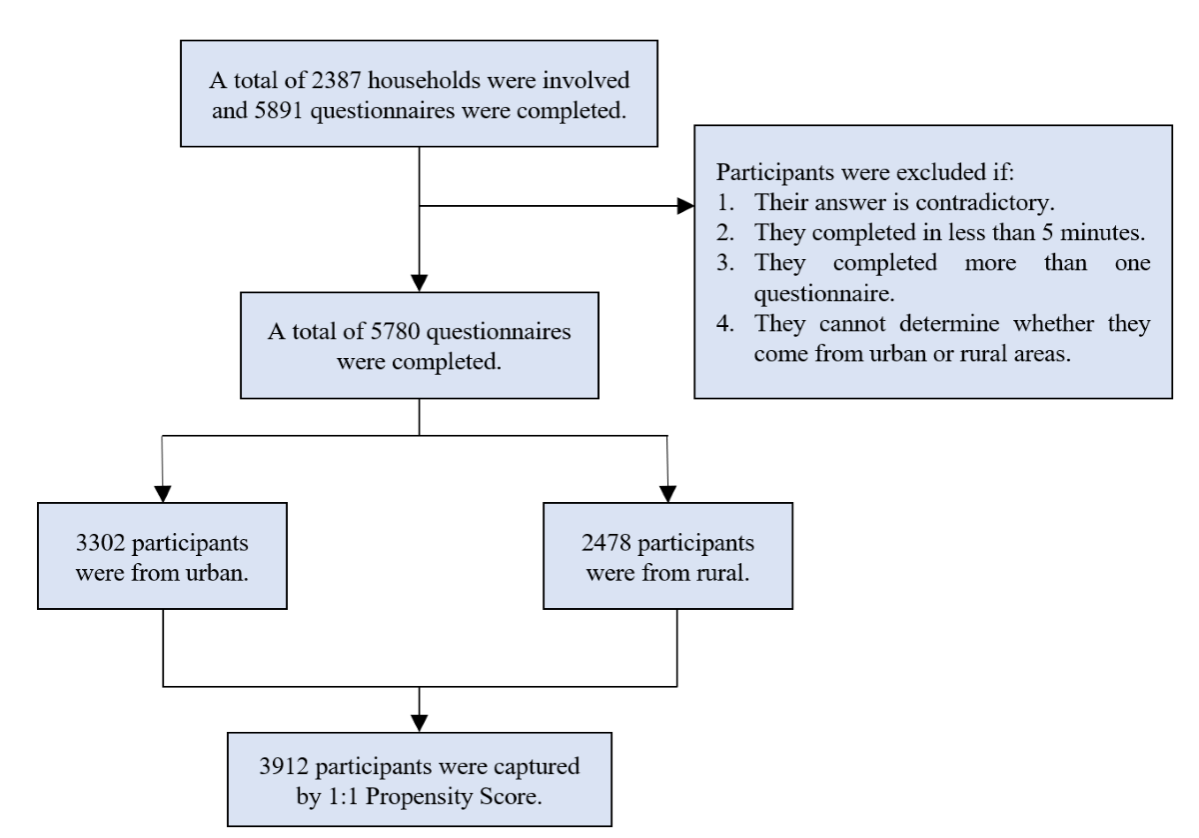
The study flowchart.

From February 1 to February 18, 2023, we selected 11 communities and 10 villages in eastern, central, western, and northeastern Mainland Chinese as representative samples using stratified random sampling. Within each region, namely Changzhou, Zhengzhou, Xining, and Mudanjiang, a random sampling approach was employed to select representative urban and rural areas. Specifically, 2 or more communities and villages were randomly chosen from each city. Furthermore, to ensure comprehensive coverage, households were also randomly selected within each region. Finally, all members of the selected family (aged ≥18 years) participated in the survey and completed a web-based or paper-based questionnaire with the investigator’s assistance. To ensure data consistency and reliability, questionnaires that contained contradictory responses to key questions were excluded. Additionally, questionnaires completed in less than 5 minutes were excluded to maintain an adequate level of response detail. Furthermore, to prevent duplicate entries and potential bias in the analysis, questionnaires that were filled out repeatedly by the same individuals were excluded. Finally, questionnaires that lacked clear identification of the survey object's source were excluded to ensure transparency and accuracy in the data collection process. Following the exclusion criteria, the questionnaires underwent a thorough review process conducted by trained staff. From this review, a total of 5891 questionnaires were collected. Subsequently, after further screening and data validation, a final sample of 5780 participants was included in our study. Figure 1 shows the study flowchart.

### Measures of Variables

Experienced epidemiologists developed the questionnaires, which contained the following components: sociodemographic information (sex, age, religion, marital status, educational level, career, social status in China, and social status in community), health conditions (chronic disease, history of allergy, self-reported health condition, and COVID-19 infection), vaccine-related information (vaccine knowledge and vaccine accessibility), information related to the Protective Motivation Theory (PMT; severity, susceptibility, benefits, barriers and self-efficacy), and the level of trust in the health care system (trust in doctors and vaccine developers).

We assigned a unique code to each participant, which was used to distinguish whether the participant came from an urban or a rural area.

COVID-19 vaccination status was measured based on participants’ responses to 3 items. The first item asked, “Have you been vaccinated with the COVID-19 basic vaccine?” We set 3 answers: 1 (“Yes, I have completed COVID-19 basic vaccination”) 2 (“Yes, but I haven't completed COVID-19 basic vaccination), and 3 (“No). The second question was, “Have you been vaccinated with the third COVID-19 (first booster) vaccine?” We set 2 answers: 1 (“Yes”) and 2 (“No”). The third question was, “Have you been vaccinated with the fourth COVID-19 (second booster) vaccine?” We set 2 answers: 1 (“Yes”) and 2 (“No”). We defined participants who completed all 3 items as those who had been vaccinated with the fourth COVID-19 (second booster) vaccine.

### Data Analysis Procedure

An independent sample *t* test or chi-square test was carried out to test the differences in COVID-19 vaccination across groups between the urban and rural participants. Binary logistic regression models were performed to explore the factors influencing vaccination among the urban and rural participants. Both univariate and multivariate analyses for urban and rural participants were conducted separately. PSM was used to minimize potential confounding biases. A probit regression model was used to estimate the propensity scores for urban and rural participants. Finally, 1956 pairs of homogeneous participants were matched in a 1:1 ratio using propensity scores from a total of 5780 participants. Differences were deemed statistically significant if *P*＜.05. We performed all statistical analyses using Stata software (version 16.1; StataCorp).

### Ethical Considerations

This study was reviewed and approved by the Life Science Ethics Review Committee of Zhengzhou University (2021-01-12-05). Written informed consent outlining the study’s objectives was obtained from each participant before the survey commenced. All data were used solely for research purposes. The study data are anonymous, and the answers are protected by privacy laws.

## Results

### Prevalence Of COVID-19 Vaccination and Characteristics Among Urban and Rural Participants

A total of 5780 participants, among them 57.13% (3302/5780) urban residents, completed the survey. A summary of COVID-19 vaccination rates, along with characteristics like sociodemographic information, health conditions, vaccine-related information, information related to PMT, and trust in the health care system between the urban and rural participants is shown in Table S1 in Multimedia Appendix 1. In the total sample, 12.18% (704/5780) of participants (95% CI 11.34-13.02) expressed they had been vaccinated with the fourth COVID-19 (second booster) vaccine. Additionally, 10.99% (363/3302) of urban participants (95% CI 9.93-12.06) had a relatively lower COVID-19 vaccination rate than rural participants at 13.76% (341/2478; 95% CI 12.40-15.12). Individuals aged 30 to 39 years, without chronic disease or previous COVID-19 infection, who had a higher subjective community level, higher level of vaccine knowledge, higher perceived benefits, higher self-efficacy, and trust doctors and vaccine developers (at levels 1 and 4) were more likely to receive a COVID-19 vaccination in urban areas (all *P*<.05). Conversely, individuals aged 18 to 29 years, without chronic disease, with vaccine accessibility within 15 minutes, higher self-reported health condition, lower level of vaccine knowledge, lower perceived benefits, higher perceived barriers, lower self-efficacy, and lower trust in doctors and vaccine developers were more likely to receive a COVID-19 vaccination in rural areas (all *P*<.05).

Additionally, we discovered that rural participants tended to be older and less educated, with fewer reporting a history of COVID-19 infection compared to urban participants. In both rural and urban participants, the proportion of female (n=1488, 45.06%) and male (n=1226, 49.48%) participants was relatively similar. Moreover, 45.34% (n=1497) of urban participants and 38.58% (n=956) of rural participants took less than 15 minutes to reach the vaccination site (Table S2 in Multimedia Appendix 1).

### Factors Influencing COVID-19 Vaccination Among Urban and Rural Participants

After adjusting for potential confounding variables, we discovered that urban participants without chronic disease (adjusted odds ratio [AOR] 1.629, 95% 1.108-2.396), without COVID-19 infection (AOR 1.977, 95% CI 1.977-2.479), who had a higher subjective community level (AOR 1.073, 95% CI 1.013-1.136), level 2 self-efficacy (AOR 2.162, 95% CI 1.550-3.014), and level 3 self-efficacy (AOR 1.842, 95% CI 1.120-3.029) had a higher COVID-19 vaccination rate. Participants from urban areas who had level 4 vaccine knowledge (AOR 0.667,95% CI 0.476-0.935), level 2 trust in doctors (AOR 0.530: 95% CI 0.377-0.743), and level 3 trust in vaccine developers (AOR 0.638, 95% CI 0.425-0.959) had a lower COVID-19 vaccination rate. The results showed that rural participants who had a higher self-reported health condition (AOR 1.009, 95% CI 1.001-1.017), level 2 self-efficacy (AOR 2.524, 95% CI 1.753-3.633), and level 3 self-efficacy (AOR 4.162, 95% CI 2.369-7.315) had a higher COVID-19 vaccination rate. Participants from rural areas who had a university degree or above (AOR 0.619, 95% CI 0.427-0.898), level 2 vaccine knowledge (AOR 0.680, 95% CI 0.501-0.923), level 4 vaccine knowledge (AOR 0.522, 95% CI 0.351-0.776), level 2 susceptibility (AOR 0.591, 95% CI 0.416-0.840), level 2 benefits (AOR 0.537, 95% CI 0.376-0.765), level 3 benefits (AOR 0.539, 95% CI 0.312-0.933), level 2 trust in doctors (AOR 0.665, 95% CI 0.456-0.969), level 3 trust in doctors (AOR 0.545, 95% CI 0.340-0.874), level 4 trust in doctors (AOR 0.364, 95% CI 0.200-0.661), and level 4 trust in vaccine developers (AOR 0.489, 95% CI 0.253-0.945) had a lower COVID-19 vaccination rate (Table S3 in Multimedia Appendix 1).

### Propensity Score Matching Analysis

**Figure 2:**
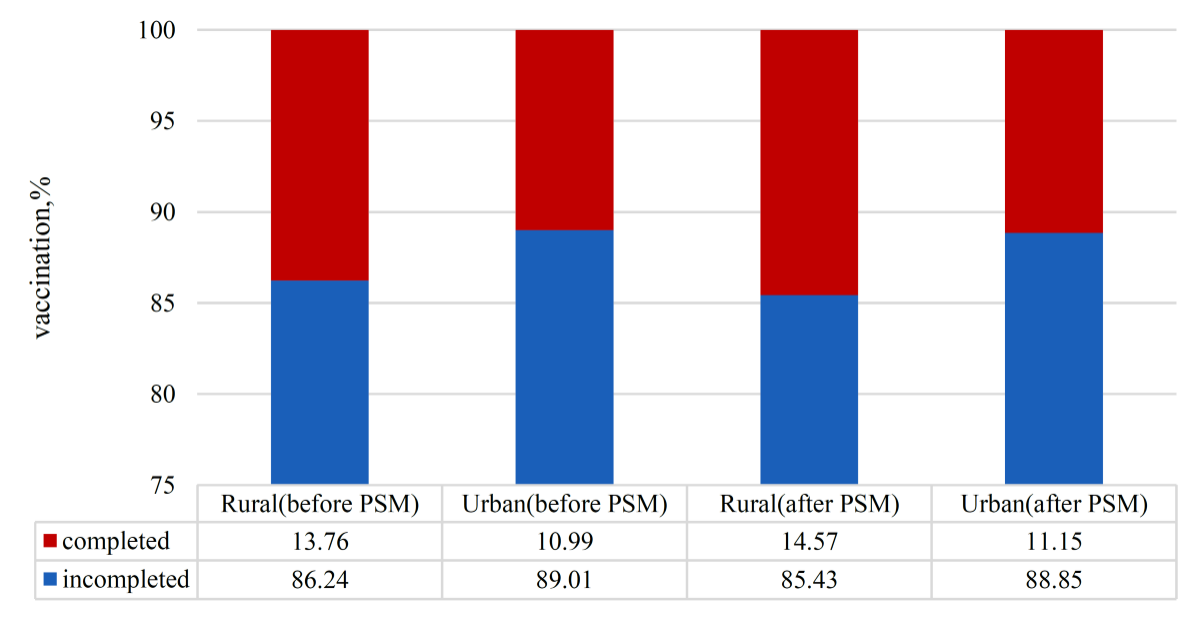
COVID-19 vaccination prevalence between urban and rural Chinese populations before and after propensity score matching (PSM).

**Figure 3:**
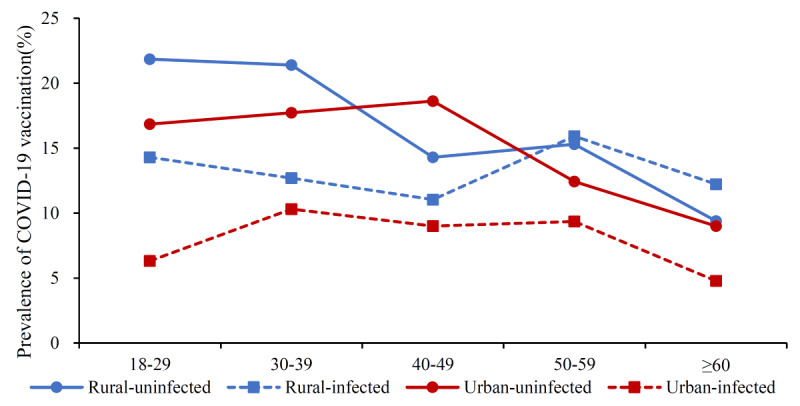
The prevalence of COVID-19 vaccination rate in all age groups by infection status of COVID-19 between urban and rural Chinese populations after propensity score matching (PSM).

In total, 3912 samples were captured using propensity score matching (PSM) from the 5780 participants. After PSM for sex, age, religion, career, self-reported health condition, educational level, COVID-19 infection, vaccine accessibility, vaccine knowledge, susceptibility, barriers, trust in doctors, and trust in vaccine developers, no statistically significant discrepancies were discerned between the urban and rural participants in all covariates (all *P>*.05). The balance test and common support domain of PSM for urban and rural samples are shown in Table S4 and Figure S1 in Multimedia Appendix 1. Based on the balanced samples, differences in COVID-19 vaccination rates among the urban and rural participants were assessed. As shown in Figure 2, the prevalence of COVID-19 vaccination among rural participants (14.57%; 95% CI 13.01-16.14) was still higher than that among urban participants (11.15%; 95% CI 9.75-12.54) by 3.42% (*P*<.05) after PSM. In addition, the vaccination rate of COVID-19 in uninfected participants was higher than that of participants who were infected in most age groups (Figure 3).

## Discussion

### Principal Findings

This study showed that 12.18% (704/5780) participants completed the fourth COVID-19 (second booster) vaccination, with 10.99% (363/3302) urban participants and 13.76% (341/2478) rural participants completing the fourth COVID-19 (second booster) vaccination, respectively. Vaccine knowledge, self-efficacy, and trust in the health care system had an impact on vaccination rates among both urban and rural residents. COVID-19 infection, chronic disease, and subjective community level had an impact on the vaccination rate among urban residents. Educational level, self-reported health condition, susceptibility, and benefits had an impact on the vaccination rate among rural residents. According to the PSM analysis, a 42% disparity in vaccine coverage rates was confirmed between urban and rural participants. In addition, the vaccination rate in uninfected participants was higher than that of participants who were infected in most age groups.

Our study unveiled differences in vaccination coverage rates between urban and rural residents, with rural residents having a higher COVID-19 vaccination rate than urban residents. This finding is consistent with those of previous studies, not only for COVID-19 but also for other vaccines [35-37]. Prior research has shown that the vaccination rate in rural areas is higher than that in urban areas [38,39]. In a survey of men who have sex with men in England, the vaccination rate for human papillomavirus (HPV) was higher in rural areas than in urban areas [38]. A COVID-19 vaccination coverage survey conducted among US veterans showed that the vaccination rate of rural veterans was higher than that of urban veterans [39]. On the other hand, some research results showed higher vaccination rates in urban areas compared to rural areas [40,41]. In a survey of COVID-19 vaccination in the United States, the findings showed that the vaccination rate in urban areas was higher compared to rural areas [40]. In a survey of influenza vaccination among adults in the United States, results showed that the vaccination rate was higher in cities than in rural areas [36]. A survey conducted among adolescents in the United States assessing the use of vaccination services for vaccines, such as MenACWY (meningococcal serogroups A, C, W, and Y), Tdap (tetanus, diphtheria, pertussis), HPV, and influenza, revealed that urban adolescents had a higher rate of utilization compared to their rural counterparts [41].

Differences in vaccination rates between urban and rural areas can be influenced by many factors [42]. We believe that there are several reasons that the rural residents had a higher COVID-19 vaccination rate than their urban counterparts. First, due to the unbalanced allocation of medical resources, rural residents have a higher mortality rate related to COVID-19, and they are more willing to be vaccinated for their own safety [43,44]. Second, with the development of urbanization in China, rural population mobility and the increase in migrant workers have led to a smaller rural population base, resulting in a higher vaccination rate [45]. Third, the Chinese government has begun paying more attention to health issues faced by the rural older adult population and has taken relevant measures to ensure that every rural older adult is vaccinated [46]. Finally, the decision to vaccinate is also easily influenced by information found on the internet. Because rural residents have lower education levels, they have higher trust in web-based information and a stronger intention to vaccinate than urban residents [47-49].

Our results showed that several factors, including vaccine knowledge level, self-efficacy, trust in medical staff, and trust in vaccine developers had an impact on the decision to vaccinate among urban and rural residents. Studies have shown that an individual's comprehension of vaccine knowledge influences their decision to get vaccinated [50,51]. Individuals who lack sufficient vaccine knowledge may be hesitant about vaccination [52]. The same applies to other types of vaccines, such as influenza vaccines, where individuals with higher levels of vaccine knowledge are more likely to receive the influenza vaccine [53]. This is similar to the results of other studies showing that an individual's self-efficacy influences their decision to receive vaccinations. Higher self-efficacy encourages individuals to get vaccinated [54,55]. Similarly, trust in doctors and vaccine developers also affects an individual's decision to get vaccinated [56,57]. A survey in Germany showed that trust in the medical establishment impacts the intention to receive the COVID-19 vaccine [58]. Confidence in public service delivery also leads to favorable responses to mass immunization efforts [59].

In summary, when formulating a vaccination plan, it is important to focus on early promotion and publicity, highlighting the necessity, safety, effectiveness, and other relevant information about the vaccine. In addition, emphasis should be placed on improving residents' self-efficacy and on doctor-patient communication to increase vaccination rates [60].

Furthermore, our analysis revealed that in both urban and rural areas, the rate of fourth COVID-19 (second booster) vaccination was generally higher among residents without a history of infection compared to those who had been previously infected with COVID-19. SARS-CoV-2 constantly changes through mutations that generate variants [61]. A study showed that the antibody response produced after vaccination better neutralizes certain prevalent variants [62]. A statistically significant decreased risk for reinfection was found among individuals who were previously infected and then vaccinated versus those who were previously infected but remained unvaccinated [63]. In addition, there was a decreased risk for symptomatic disease among previously infected and vaccinated persons compared with those who were not vaccinated after infection [64]. Therefore, even if residents have a history of infection, they should be vaccinated again to safeguard against future infections. We suggest that when promoting booster vaccinations, it should be emphasized that individuals should receive the booster even if they have been infected with SARS-CoV-2 and have acquired antibodies.

### Strengths and Limitations

This study was the first nationwide survey on the vaccination status of residents in China during the fourth COVID-19 (second booster) vaccination campaign. Given the complexity of factors influencing COVID-19 vaccination, we used PSM for the first time to mitigate confounding variables and quantify disparities in COVID-19 vaccination rates in rural and urban areas.

Additionally, this study used 1:1 matching with PSM, highlighting 2 advantages. First, the PSM method ensured that all indicators were collected from homogeneous respondents from both urban and rural areas. Second, 1:1 matching maximized the utilization of group information to ensure that individuals within each group were closely matched, enhancing the strength of the principal findings.

However, this study has numerous limitations that must be acknowledged. First, the assessment of COVID-19 vaccination relied on self-reported questionnaires, which inevitably introduced subjectivity, self-bias, and response bias in the data collection. Second, while the study employed the PMT framework to comprehensively collect COVID-19 vaccination covariates, it is possible that some unknown related factors were not collected.

### Conclusion

The fourth COVID-19 (second booster) vaccination coverage rate among the Chinese population was exceptionally low, significantly lower than previous vaccine coverage rates. With COVID-19 infections still prevalent at a low level, efforts should be concentrated on enhancing self-efficacy and building trust in the health care system to expand vaccine coverage among the Chinese population. For rural residents, building confidence in vaccination benefits and improving overall health status must be prioritized. In urban areas, focusing on vaccinating a larger proportion of people with COVID-19 and patients with chronic disease is crucial. In conclusion, the vaccination rate is higher in rural areas than in urban areas. Therefore, attention should be given to urban-rural differences and targeted vaccination plans should be formulated when implementing vaccination programs. The results of this study offer valuable insights for future responses to emerging major infectious diseases.

## Appendix

Multimedia Appendix 1 Additional Tables and Figures.

## Abbreviations

AOR: adjusted odds ratio
HPV: human papillomavirus
Men ACWY: meningococcal serogroups A, C, W, and Y
PMT: Protective Motivation Theory
PSM: propensity score matching
Tdap: tetanus, diphtheria, pertussis

## Data Availability

All data can be acquired by contacting the corresponding author.
