# Supplementary material for: Quantifying Disparities in COVID-19 Vaccination Rates by Rural and Urban Areas: Cross-Sectional Observational Study
Source: JMIR Public Health Surveill. 2024 Jul 19;10:e50595. doi: 10.2196/50595 (PMC11297372; doi:10.2196/50595)
Supplement: Multimedia Appendix 1 [file publichealth_v10i1e50595_app1.docx]

**Appendix：**

**Table S1. The characteristics and overall COVID-19 vaccination of all study participants** **from February 1 to February 18, 2023 in China(n=5780).**

| Covariates | | Total | | Urban | | | Rural | | | |
| --- | --- | --- | --- | --- | --- | --- | --- | --- | --- | --- |
|  |  | N (%) | *P* value^a^ | N (%) | Completed  (%,95%CI) | *P* value^b^ |  | N (%) | Completed  (%,95%CI) | *P* value^c^ |
| Total | | 5780(100) |  | 3302(100) | 10.99(9.93~12.06) |  | 2478(100) | | 13.76(12.40~15.12) |  |
| Sex | |  | 0.774 |  |  | 0.195 |  | |  | 0.463 |
|  | Male | 2714(46.96) |  | 1488(45.06) | 10.22(8.67~11.76) |  | 1226(49.48) | | 14.27(12.31~16.23) |  |
|  | Female | 3066(53.04) |  | 1814(54.94) | 11.63(10.15~13.11) |  | 1252(50.52) | | 13.26(11.38~15.14) |  |
| Age | |  | 0.001 |  |  | 0.020 |  | |  | 0.009 |
|  | 18-29 | 659(11.40) |  | 369(11.18) | 10.57(7.42~13.72) |  | 290(11.70) | | 17.24(12.87~21.61) |  |
|  | 30-39 | 1556(26.92) |  | 1027(31.10) | 12.66(10.62~14.70) |  | 529(21.35) | | 16.45(13.28~19.62) |  |
|  | 40-49 | 1141(19.74) |  | 729(22.08) | 12.07(9.70~14.44) |  | 412(16.63) | | 12.62(9.40~15.84) |  |
|  | 50-59 | 1191(20.61) |  | 540(16.35) | 10.74(8.12~13.36) |  | 651(26.27) | | 14.13(11.45~16.82) |  |
|  | ≥60 | 1233(21.33) |  | 637(19.29) | 7.54(5.48~9.59) |  | 596(24.05) | | 10.07(7.64~12.49) |  |
| Religion | |  | 0.939 |  |  | 0.587 |  | |  | 0.887 |
|  | Atheist | 5514(95.40) |  | 3215(97.37) | 11.04(9.96~12.13) |  | 2299(92.78) | | 13.79(12.38~15.20) |  |
|  | Others | 266(4.60) |  | 87(2.63) | 9.20(3.00~15.39) |  | 179(7.22) | | 13.41(8.37~18.45) |  |
| Marital status | |  | 0.424 |  |  | 0.247 |  | |  | 0.816 |
|  | Married | 5112(88.44) |  | 2894(87.64) | 11.23(10.08~12.38) |  | 2218(89.51) | | 13.71(12.27~15.14) |  |
|  | Others | 668(11.56) |  | 408(12.36) | 9.31(6.48~12.15) |  | 260(10.49) | | 14.23(9.96~18.51) |  |
| Educational level | |  | 0.099 |  |  | 0.486 |  | |  | 0.186 |
|  | Below high school | 2487(43.03) |  | 1054(31.92) | 10.53(8.68~12.39) |  | 1433(57.83) | | 13.68(11.90~15.46) |  |
|  | High school | 1445(25.00) |  | 913(27.65) | 12.05(9.93~14.16) |  | 532(21.47) | | 15.79(12.68~18.90) |  |
|  | University graduate | 1848(31.97) |  | 1335(40.43) | 10.64(8.98~12.29) |  | 513(20.70) | | 11.89(9.08~14.70) |  |
| Career | |  | 0.133 |  |  | 0.313 |  | |  | 0.226 |
|  | Others | 5523(95.55) |  | 3146(95.28) | 10.87(9.78~11.96) |  | 2377(95.92) | | 13.59(12.21~14.97) |  |
|  | Medical staff | 257(4.45) |  | 156(4.72) | 13.46(8.05~18.88) |  | 101(4.08) | | 17.82(10.23~25.41) |  |
| Chronic condition | |  | ＜0.001 |  |  | ＜0.001 |  | |  | 0.002 |
|  | Yes | 1003(17.35) |  | 553(16.75) | 6.33(4.29~8.36) |  | 450(18.16) | | 9.11(6.44~11.78) |  |
|  | No | 4777(82.65) |  | 2749(83.25) | 11.93(10.72~13.14) |  | 2028(81.84) | | 14.79(13.25~16.34) |  |
| Allergy history | |  | 0.215 |  |  | 0.374 |  | |  | 0.124 |
|  | Yes | 409(7.08) |  | 282(8.54) | 8.51(5.23~11.79) |  | 127(5.13) | | 15.75(9.33~22.17) |  |
|  | No | 4766(82.46) |  | 2638(79.89) | 11.26(10.05~12.47) |  | 2128(85.88) | | 14.10(12.62~15.58) |  |
|  | Unclear | 605(10.47) |  | 382(11.57) | 10.99(7.84~14.15) |  | 223(9.00) | | 9.42(5.55~13.28) |  |
| COVID-19 infection | |  | ＜0.001 |  |  | ＜0.001 |  | |  | 0.395 |
|  | Yes | 3165(54.76) |  | 1913(57.93) | 8.21(6.98~9.44) |  | 1252(50.52) | | 13.18(11.30~15.06) |  |
|  | No | 2615(45.24) |  | 1389(42.07) | 14.83(12.96~16.70) |  | 1226(49.48) | | 14.36(12.39~16.32) |  |
| Vaccine accessibility | |  | 0.107 |  |  | 0.413 |  | |  | 0.003 |
|  | ＜15m | 2453(42.44) |  | 1497(45.34) | 11.29(9.68~12.89) |  | 956(38.58) | | 15.06(12.79~17.33) |  |
|  | 15-30m | 2569(44.45) |  | 1423(43.10) | 10.19(8.62~11.76) |  | 1146(46.25) | | 12.83(10.89~14.77) |  |
|  | ＞30m | 436(7.54) |  | 163(4.94) | 14.11(8.71~19.51) |  | 273(11.02) | | 9.52(6.02~13.03) |  |
|  | Unclear | 322(5.57) |  | 219(6.63) | 11.87(7.55~16.19) |  | 103(4.16) | | 23.30(15.00~31.60) |  |
| Self-report health condition | | 80.83(80.42~81.24) | ＜0.001 | 80.38(79.82~80.95) ^d^ | 81.23(79.42~83.03) ^d^ | 0.099 | 81.42(80.83~82.01) ^d^ | | 83.30(81.47~85.12) ^d^ | ＜0.001 |
| Subjective social level | | 5.00(4.94~5.05) | 0.137 | 5.07(5.00~5.13) ^d^ | 5.27(5.04~5.49) ^d^ | 0.078 | 4.90(4.81~4.99) ^d^ | | 5.02(4.77~5.27) ^d^ | 0.574 |
| Subjective community status | | 5.05(5.00~5.11) | 0.026 | 5.13(5.06~5.20) ^d^ | 5.37(5.15~5.60) ^d^ | 0.016 | 4.95(4.86~5.04) ^d^ | | 5.12(4.87~5.37) ^d^ | 0.315 |
| Vaccine knowledge | |  | ＜0.001 |  |  | 0.001 |  | |  | ＜0.001 |
|  | Level1 | 1872(32.39) |  | 1111(33.65) | 10.71(8.89~12.53) |  | 761(30.71) | | 19.32(16.51~22.13) |  |
|  | Level2 | 1551(26.83) |  | 839(25.41) | 11.92(9.72~14.12) |  | 712(28.73) | | 13.20(10.71~15.69) |  |
|  | Level3 | 1058(18.30) |  | 535(16.20) | 14.77(11.75~17.78) |  | 523(21.11) | | 11.28(8.56~14.00) |  |
|  | Level4 | 1299(22.47) |  | 817(24.74) | 7.96(6.10~9.82) |  | 482(19.45) | | 8.51(6.01~11.01) |  |
| Severity | |  | 0.736 |  |  | 0.591 |  | |  | 0.136 |
|  | Level1 | 2791(48.29) |  | 1688(51.12) | 11.14(9.64~12.64) |  | 1103(44.51) | | 14.69(12.59~16.78) |  |
|  | Level2 | 515(8.91) |  | 320(9.69) | 11.25(7.77~14.73) |  | 195(7.87) | | 15.38(10.28~20.49) |  |
|  | Level3 | 1667(28.84) |  | 955(28.92) | 10.05(8.14~11.96) |  | 712(28.73) | | 14.04(11.49~16.60) |  |
|  | Level4 | 807(13.96) |  | 339(10.27) | 12.68(9.12~16.25) |  | 468(18.89) | | 10.47(7.69~13.25) |  |
| Susceptibility | |  | 0.146 |  |  | 0.147 |  | |  | 0.242 |
|  | Level1 | 1450(25.09) |  | 934(28.29) | 11.03(9.02~13.04) |  | 516(20.82) | | 15.89(12.73~19.06) |  |
|  | Level2 | 2071(35.83) |  | 1211(36.67) | 12.30(10.45~14.16) |  | 860(34.71) | | 14.19(11.85~16.52) |  |
|  | Level3 | 1013(17.53) |  | 616(18.66) | 8.77(6.53~11.01) |  | 397(16.02) | | 13.35(9.99~16.71) |  |
|  | Level4 | 1246(21.56) |  | 541(16.38) | 10.54(7.94~13.13) |  | 705(28.45) | | 11.91(9.52~14.31) |  |
| Benefits | |  | 0.009 |  |  | ＜0.001 |  | |  | ＜0.001 |
|  | Level1 | 1908(33.01) |  | 1221(36.98) | 10.57(8.84~12.29) |  | 687(27.72) | | 19.94(16.95~22.94) |  |
|  | Level2 | 2869(49.64) |  | 1652(50.03) | 9.87(8.43~11.31) |  | 1217(49.11) | | 12.49(10.63~14.35) |  |
|  | Level3 | 1003(17.35) |  | 429(12.99) | 16.55(13.02~20.08) |  | 574(23.16) | | 9.06(6.70~11.41) |  |
| Barriers | |  | ＜0.001 |  |  | 0.228 |  | |  | ＜0.001 |
|  | Level1 | 2436(42.15) |  | 1193(36.13) | 10.90(9.13~12.67) |  | 1243(50.16) | | 9.98(8.31~11.64) |  |
|  | Level2 | 530(9.17) |  | 321(9.72) | 7.79(4.84~10.74) |  | 209(8.43) | | 8.61(4.78~12.45) |  |
|  | Level3 | 1998(34.57) |  | 1261(38.19) | 11.42(9.66~13.18) |  | 737(29.74) | | 18.86(16.03~21.69) |  |
|  | Level4 | 816(14.12) |  | 527(15.96) | 12.14(9.35~14.94) |  | 289(11.66) | | 20.76(16.06~25.47) |  |
| Self-efficacy | |  | 0.033 |  |  | 0.001 |  | |  | 0.012 |
|  | Level1 | 1914(33.11) |  | 1202(36.40) | 8.65(7.06~10.24) |  | 712(28.73) | | 14.04(11.49~16.60) |  |
|  | Level2 | 2758(47.72) |  | 1627(49.27) | 11.62(10.06~13.18) |  | 1131(45.64) | | 15.47(13.36~17.58) |  |
|  | Level3 | 1108(19.17) |  | 473(14.32) | 14.80(11.59~18.01) |  | 635(25.63) | | 10.39(8.01~12.77) |  |
| Trust in doctors | |  | ＜0.001 |  |  | ＜0.001 |  | |  | ＜0.001 |
|  | Level1 | 1564(27.06) |  | 991(30.01) | 13.52(11.39~15.65) |  | 573(23.12) | | 22.51(19.08~25.94) |  |
|  | Level2 | 1895(32.79) |  | 1182(35.80) | 8.38(6.79~9.96) |  | 713(28.77) | | 15.01(12.38~17.63) |  |
|  | Level3 | 989(17.11) |  | 547(16.57) | 9.51(7.04~11.97) |  | 442(17.84) | | 11.99(8.95~15.03) |  |
|  | Level4 | 1332(23.04) |  | 582(17.63) | 13.40(10.63~16.18) |  | 750(30.27) | | 6.93(5.11~8.76) |  |
| Trust in vaccine developers | |  | ＜0.001 |  |  | ＜0.001 |  | |  | ＜0.001 |
|  | Level1 | 2065(35.73) |  | 1320(39.98) | 12.12(10.36~13.88) |  | 745(30.06) | | 19.33(16.49~22.17) |  |
|  | Level2 | 1012(17.51) |  | 665(20.14) | 10.23(7.92~12.53) |  | 347(14.00) | | 20.46(16.20~24.73) |  |
|  | Level3 | 1512(26.16) |  | 826(25.02) | 7.63(581~9.44) |  | 686(27.68) | | 11.66(9.25~14.07) |  |
|  | Level4 | 1191(20.61) |  | 491(14.87) | 14.66(11.52~17.80) |  | 700(28.25) | | 6.57(4.73~8.41) |  |

Note:

CI, confidence interval.

COVID-19, Coronavirus disease 2019.

^a^ Differences between categories within each variable in total samples.

^b^ Differences between categories within each variable in urban samples.

^c^ Differences between categories within each variable in rural samples.

^d^ Mean and 95% confidence interval for variables.

We categorized the score of vaccine knowledge by quartiles as level 1 (1-2 points), level 2 (3-4 points), level 3 (5-6 points), and level 4 (7-9 points) , the score of severity by quartiles as level 1 (3-9 points), level 2 (10 points), level 3 (11-12 points), and level 4 (13-15 points), the score of susceptibility by quartiles as level 1 (3-7 points), level 2 (8-9 points), level 3 (10-11 points), and level 4 (12-15points), the score of benefits by quartiles as level 1 as (3-9 points), level 2 (10-12 points), and level 3 (13-15 points), the score of barriers by quartiles as level 1 as (4-8 points), level 2 (9-10 points), level 3 (11-12 points), and level 4 (13-20 points), the score of self-efficacy by quartiles as level 1 as (4-12 points), level 2 (13-16 points), and level 3 (17-20 points), the score of trust in doctors by quartiles as level 1(11-29 points), level 2 (30-34 points), level 3 (35-36 points), and level 4 (37-45 points) and the score of trust in vaccine developers by quartiles as level 1 (5-15 points), level 2 (16-19 points), level 3 (20 points), and level 4 (21-25 points).

**Table S2. Associations between COVID-19 vaccination and sociodemographic, health condition, vaccine related information, severity, susceptibility, benefits, barriers, self-efficiency and trust in health care system in urban and rural areas of all participants from February 1 to February 18, 2023 in China(n=5780).**

| Covariates | | Urban | | | | Rural | | | | |
| --- | --- | --- | --- | --- | --- | --- | --- | --- | --- | --- |
|  |  | Model 1 | *P* | Model 2 | *P* |  | Model 1 | *P* | Model 2 | *P* |
| Sex | |  |  |  |  |  | |  |  |  |
|  | Male | 1.00(ref.) |  | 1.00(ref.) |  | 1.00(ref.) | |  | 1.00(ref.) |  |
|  | Female | 1.157(0.928~1.443) | 0.196 | 1.140(0.908~1.431) | 0.258 | 0.918(0.730~1.154) | | 0.463 | 0.932(0.734~1.182) | 0.560 |
| Age | |  |  |  |  |  | |  |  |  |
|  | 18-29 | 1.00(ref.) |  | 1.00(ref.) |  | 1.00(ref.) | |  | 1.00(ref.) |  |
|  | 30-39 | 1.226(0.839~1.792) | 0.292 | 1.352(0.916~1.997) | 0.129 | 0.945(0.645~1.384) | | 0.771 | 0.840(0.562~1.255) | 0.394 |
|  | 40-49 | 1.162(0.779~1.733) | 0.463 | 1.240(0.822~1.870) | 0.305 | 0.693(0.455~1.056) | | 0.088 | 0.724(0.466~1.125) | 0.151 |
|  | 50-59 | 1.018(0.663~1.564) | 0.934 | 1.214(0.779~1.893) | 0.392 | 0.790(0.542~1.151) | | 0.219 | 0.929(0.623~1.384) | 0.716 |
|  | ≥60 | 0.690(0.443~1.074) | 0.100 | 0.890(0.559~1.418) | 0.625 | 0.537(0.358~0.806) | | 0.003 | 0.687(0.441~1.070) | 0.097 |
| Religion | |  |  |  |  |  | |  |  |  |
|  | Atheist | 1.00(ref.) |  | 1.00(ref.) |  | 1.00(ref.) | |  | 1.00(ref.) |  |
|  | Others | 0.816(0.391~1.702) | 0.588 | 0.824(0.386~1.760) | 0.617 | 0.968(0.620~1.512) | | 0.887 | 0.780(0.485~1.253) | 0.304 |
| Marriage | |  |  |  |  |  | |  |  |  |
|  | Married | 1.00(ref.) |  | 1.00(ref.) |  | 1.00(ref.) | |  | 1.00(ref.) |  |
|  | Others | 0.812(0.570~1.156) | 0.247 | 0.629(0.385~1.027) | 0.064 | 1.045(0.723~1.510) | | 0.816 | 0.873(0.531~1.435) | 0.592 |
| Educational level | | |  |  |  |  | |  |  |  |
|  | Below high school | 1.00(ref.) |  | 1.00(ref.) |  | 1.00(ref.) | |  | 1.00(ref.) |  |
|  | High school | 1.164(0.880~1.540) | 0.288 | 0.974(0.722~1.315) | 0.865 | 1.183(0.897~1.562) | | 0.234 | 0.970(0.716~1.314) | 0.846 |
|  | University | 1.011(0.778~1.315) | 0.934 | 0.847(0.617~1.163) | 0.304 | 0.852(0.627~1.158) | | 0.305 | 0.619(0.427~0.898) | 0.011 |
| Career | |  |  |  |  |  | |  |  |  |
|  | Others | 1.00(ref.) |  | 1.00(ref.) |  | 1.00(ref.) | |  | 1.00(ref.) |  |
|  | Medical staff | 1.275(0.795~1.047) | 0.314 | 1.373(0.836~2.254) | 0.210 | 1.379(0.817~2.326) | | 0.228 | 1.536(0.882~2.675) | 0.129 |
| Chronic condition | |  |  |  |  |  | |  |  |  |
|  | Yes | 1.00(ref.) |  | 1.00(ref.) |  | 1.00(ref.) | |  | 1.00(ref.) |  |
|  | No | 2.005(1.397~2.877) | ＜0.001 | 1.626(1.106~2.391) | 0.013 | 1.732(1.228~2.442) | | 0.002 | 1.363(0.928~2.001) | 0.114 |
| Allergy history | |  |  |  |  |  | |  |  |  |
|  | Yes | 1.00(ref.) |  | 1.00(ref.) |  | 1.00(ref.) | |  | 1.00(ref.) |  |
|  | No | 1.364(0.882~2.108) | 0.162 | 1.238(0.791~1.937) | 0.350 | 0.878(0.536~1.437) | | 0.605 | 0.926(0.548~1.566) | 0.774 |
|  | Unclear | 1.328(0.784~2.249) | 0.291 | 1.333(0.773~2.299) | 0.302 | 0.556(0.289~1.071) | | 0.079 | 0.578(0.290~1.152) | 0.119 |
| COVID-19 infection | | |  |  |  |  | |  |  |  |
|  | Yes | 1.00(ref.) |  | 1.00(ref.) |  | 1.00(ref.) | |  | 1.00(ref.) |  |
|  | No | 1.948(1.562~2.428) | ＜0.001 | 1.976(1.576~2.477) | ＜0.001 | 1.104(0.879~1.388) | | 0.395 | 1.033(0.810~1.318) | 0.794 |
| Vaccine accessibility | | |  |  |  |  | |  |  |  |
|  | ＜15m | 1.00(ref.) |  | 1.00(ref.) |  | 1.00(ref.) | |  | 1.00(ref.) |  |
|  | 15-30m | 0.892(0.705~1.127) | 0.338 | 0.939(0.736~1.197) | 0.611 | 0.830(0.648~1.063) | | 0.140 | 0.963(0.742~1.250) | 0.776 |
|  | ＞30m | 1.291(0.808~2.064) | 0.286 | 1.362(0.836~2.219) | 0.214 | 0.594(0.382~0.923) | | 0.021 | 0.924(0.575~1.484) | 0.743 |
|  | Unclear | 1.059(0.682~1.643) | 0.800 | 0.959(0.607~1.517) | 0.859 | 1.713(1.049~2.796) | | 0.031 | 1.333(0.787~2.260) | 0.285 |
| Self-report health condition | | 1.004(0.997~1.010) | 0.303 | 0.999(0.992~1.006) | 0.766 | 1.010(1.002~1.019) | | 0.012 | 1.009(1.001~1.017) | 0.036 |
| Subjective social level | | 1.062(1.004~1.124) | 0.036 | 0.931(0.803~1.078) | 0.339 | 1.029(0.977~1.083) | | 0.282 | 0.985(0.929~1.043) | 0.603 |
| Subjective community level | | 1.075(1.016~1.138) | 0.012 | 1.073(1.013~1.136) | 0.016 | 1.041(0.988~1.096) | | 0.130 | 0.996(0.940~1.056) | 0.905 |
| Vaccine knowledge | | |  |  |  |  | |  |  |  |
|  | Level1 | 1.00(ref.) |  | 1.00(ref.) |  | 1.00(ref.) | |  | 1.00(ref.) |  |
|  | Level2 | 1.128(0.850~1.496) | 0.403 | 1.017(0.756~1.369) | 0.907 | 0.635(0.479~0.842) | | 0.002 | 0.680(0.501~0.923) | 0.013 |
|  | Level3 | 1.444(1.064~1.960) | 0.018 | 1.209(0.871~1.678) | 0.256 | 0.531(0.384~0.735) | | ＜0.001 | 0.704(0.491~1.007) | 0.055 |
|  | Level4 | 0.721(0.525~0.989) | 0.043 | 0.666(0.475~0.933) | 0.019 | 0.388(0.269~0.561) | | ＜0.001 | 0.522(0.351~0.776) | 0.001 |
| Severity | |  |  |  |  |  | |  |  |  |
|  | Level1 | 1.00(ref.) |  | 1.00(ref.) |  | 1.00(ref.) | |  | 1.00(ref.) |  |
|  | Level2 | 1.011(0.693~1.477) | 0.953 | 1.027(0.692~1.524) | 0.895 | 1.056(0.692~1.613) | | 0.800 | 1.037(0.658~1.636) | 0.875 |
|  | Level3 | 0.892(0.688~1.156) | 0.387 | 0.971(0.736~1.282) | 0.837 | 0.949(0.725~1.242) | | 0.704 | 1.124(0.825~1.532) | 0.458 |
|  | Level4 | 1.159(0.814~1.651) | 0.414 | 1.052(0.712~1.553) | 0.800 | 0.679(0.484~0.954) | | 0.026 | 1.198(0.763~1.882) | 0.433 |
| Susceptibility | |  |  |  |  |  | |  |  |  |
|  | Level1 | 1.00(ref.) |  | 1.00(ref.) |  | 1.00(ref.) | |  | 1.00(ref.) |  |
|  | Level2 | 1.132(0.867~1.478) | 0.363 | 1.284(0.956~1.724) | 0.097 | 0.875(0.646~1.186) | | 0.389 | 0.591(0.416~0.840) | 0.003 |
|  | Level3 | 0.775(0.548~1.096) | 0.150 | 0.918(0.636~1.325) | 0.648 | 0.815(0.561~1.184) | | 0.284 | 0.713(0.476~1.068) | 0.101 |
|  | Level4 | 0.950(0.675~1.338) | 0.770 | 1.060(0.733~1.531) | 0.758 | 0.716(0.516~0.994) | | 0.046 | 0.870(0.601~1.259) | 0.459 |
| Benefits | |  |  |  |  |  | |  |  |  |
|  | Level1 | 1.00(ref.) |  | 1.00(ref.) |  | 1.00(ref.) | |  | 1.00(ref.) |  |
|  | Level2 | 0.927(0.726~1.183) | 0.540 | 0.878(0.643~1.200) | 0.416 | 0.573(0.445~0.738) | | ＜0.001 | 0.537(0.376~0.765) | 0.001 |
|  | Level3 | 1.679(1.227~2.296) | 0.001 | 1.410(0.899~2.211) | 0.134 | 0.400(0.284~0.562) | | ＜0.001 | 0.539(0.312~0.933) | 0.027 |
| Barriers | |  |  |  |  |  | |  |  |  |
|  | Level1 | 1.00(ref.) |  | 1.00(ref.) |  | 1.00(ref.) | |  | 1.00(ref.) |  |
|  | Level2 | 0.691(0.442~1.080) | 0.105 | 0.753(0.468~1.211) | 0.242 | 0.850(0.507~1.427) | | 0.540 | 0.585(0.337~1.015) | 0.057 |
|  | Level3 | 1.054(0.820~1.356) | 0.681 | 1.110(0.760~1.621) | 0.588 | 2.098(1.615~2.725) | | ＜0.001 | 1.039(0.695~1.554) | 0.851 |
|  | Level4 | 1.130(0.822~1.554) | 0.451 | 1.037(0.679~1.585) | 0.866 | 2.364(1.684~3.320) | | ＜0.001 | 1.165(0.734~1.850) | 0.517 |
| Self-efficacy | |  |  |  |  |  | |  |  |  |
|  | Level1 | 1.00(ref.) |  | 1.00(ref.) |  | 1.00(ref.) | |  | 1.00(ref.) |  |
|  | Level2 | 1.388(1.079~1.785) | 0.011 | 2.162(1.551~3.015) | ＜0.001 | 1.120(0.859~1.461) | | 0.402 | 2.524(1.753~3.633) | ＜0.001 |
|  | Level3 | 1.834(1.327~2.535) | ＜0.001 | 1.843(1.121~3.030) | 0.016 | 0.710(0.510~0.989) | | 0.043 | 4.162(2.369~7.315) | ＜0.001 |
| Trust in doctors | |  |  |  |  |  | |  |  |  |
|  | Level1 | 1.00(ref.) |  | 1.00(ref.) |  | 1.00(ref.) | |  | 1.00(ref.) |  |
|  | Level2 | 0.585(0.444~0.770) | ＜0.001 | 0.529(0.377~0.742) | ＜0.001 | 0.608(0.457~0.807) | | 0.001 | 0.665(0.456~0.969) | 0.034 |
|  | Level3 | 0.672(0.479~0.943) | 0.021 | 0.670(0.435~1.030) | 0.068 | 0.469(0.331~0.664) | | ＜0.001 | 0.545(0.340~0.874) | 0.012 |
|  | Level4 | 0.990(0.733~1.336) | 0.947 | 0.629(0.387~1.022) | 0.061 | 0.256(0.182~0.361) | | ＜0.001 | 0.364(0.200~0.661) | 0.001 |
| Trust in vaccine developers | | |  |  |  |  | |  |  |  |
|  | Level1 | 1.00(ref.) |  | 1.00(ref.) |  | 1.00(ref.) | |  | 1.00(ref.) |  |
|  | Level2 | 0.826(0.612~1.115) | 0.212 | 0.839(0.590~1.193) | 0.329 | 1.074(0.781~1.476) | | 0.661 | 1.427(0.957~2.217) | 0.081 |
|  | Level3 | 0.599(0.441~0.813) | 0.001 | 0.639(0.425~0.960) | 0.031 | 0.551(0.410~0.741) | | ＜0.001 | 0.993(0.612~1.612) | 0.977 |
|  | Level4 | 1.246(0.923~1.681) | 0.151 | 1.032(0.633~1.683) | 0.900 | 0.294(0.207~0.417) | | ＜0.001 | 0.489(0.253~0.945) | 0.033 |

Note:

COVID-19, Coronavirus disease 2019.

Model 1, unadjusted.

Model 2, adjusted age, chronic condition, COVID-19 infection, vaccine accessibility, self-report health condition, subjective community status, vaccine knowledge, benefits barriers, self-efficacy, trust in doctors and trust in vaccine developers.

We categorized the score of vaccine knowledge by quartiles as level 1 (1-2 points), level 2 (3-4 points), level 3 (5-6 points), and level 4 (7-9 points) , the score of severity by quartiles as level 1 (3-9 points), level 2 (10 points), level 3 (11-12 points), and level 4 (13-15 points), the score of susceptibility by quartiles as level 1 (3-7 points), level 2 (8-9 points), level 3 (10-11 points), and level 4 (12-15points), the score of benefits by quartiles as level 1 as (3-9 points), level 2 (10-12 points), and level 3 (13-15 points), the score of barriers by quartiles as level 1 as (4-8 points), level 2 (9-10 points), level 3 (11-12 points), and level 4 (13-20 points), the score of self-efficacy by quartiles as level 1 as (4-12 points), level 2 (13-16 points), and level 3 (17-20 points), the score of trust in doctors by quartiles as level 1(11-29 points), level 2 (30-34 points), level 3 (35-36 points), and level 4 (37-45 points) and the score of trust in vaccine developers by quartiles as level 1 (5-15 points), level 2 (16-19 points), level 3 (20 points), and level 4 (21-25 points).

**Table S3. Characteristics of urban and rural participants.**

| Covariates | | Number of urban participants (%) | Number of rural participants (%) | P value |
| --- | --- | --- | --- | --- |
|  | N (%) |  |  |  |
| Total | 5780(100) | 3302(57.13) | 2478(42.87) |  |
| Sex | |  |  | 0.001 |
|  | Men | 1488(45.06) | 1226(49.48) |  |
|  | Women | 1814(54.94) | 1252(50.52) |  |
| Age | |  |  | ＜0.001 |
|  | 18-29 | 369(11.18) | 290(11.70) |  |
|  | 30-39 | 1027(31.10) | 529(21.35) |  |
|  | 40-49 | 729(22.08) | 412(16.63) |  |
|  | 50-59 | 540(16.35) | 651(26.27) |  |
|  | ≥60 | 637(19.29) | 596(24.05) |  |
| Religion | |  |  | ＜0.001 |
|  | Atheist | 3215(97.37) | 2299(92.78) |  |
|  | Others | 87(2.63) | 179(7.22) |  |
| Marital status | |  |  | 0.028 |
|  | Married | 2894(87.64) | 2218(89.51) |  |
|  | Others | 408(12.36) | 260(10.49) |  |
| Educational level | |  |  | ＜0.001 |
|  | Below high school | 1054(31.92) | 1433(57.83) |  |
|  | High school | 913(27.65) | 532(21.47) |  |
|  | University graduate | 1335(40.43) | 513(20.70) |  |
| Career | |  |  | 0.236 |
|  | Others | 3146(95.28) | 2377(95.92) |  |
|  | Medical staff | 156(4.72) | 101(4.08) |  |
| Chronic condition | |  |  | 0.161 |
|  | Yes | 553(16.75) | 450(18.16) |  |
|  | No | 2749(83.25) | 2028(81.84) |  |
| Allergy history | |  |  | ＜0.001 |
|  | Yes | 282(8.54) | 127(5.13) |  |
|  | No | 2638(79.89) | 2128(85.88) |  |
|  | Unclear | 382(11.57) | 223(9.00) |  |
| COVID-19 infection | |  |  | ＜0.001 |
|  | Yes | 1913(57.93) | 1252(50.52) |  |
|  | No | 1389(42.07) | 1226(49.48) |  |
| Vaccine accessibility | |  |  | ＜0.001 |
|  | ＜15m | 1497(45.34) | 956(38.58) |  |
|  | 15-30m | 1423(43.10) | 1146(46.25) |  |
|  | ＞30m | 163(4.94) | 273(11.02) |  |
|  | Unclear | 219(6.63) | 103(4.16) |  |
| Self-report health condition | | 80.38±16.519 | 81.42±15.855 | 0.205 |
| Subjective social level | | 5.07±1.933 | 4.90±2.206 | 0.005 |
| Subjective community status | | 5.13±1.933 | 4.95±2.199 | 0.002 |
| Vaccine knowledge | |  |  | ＜0.001 |
|  | Level1 | 1111(33.65) | 761(30.71) |  |
|  | Level2 | 839(25.41) | 712(28.73) |  |
|  | Level3 | 535(16.20) | 523(21.11) |  |
|  | Level4 | 816(24.71) | 482(19.45) |  |
| Severity | |  |  | ＜0.001 |
|  | Level1 | 1688(51.12) | 1103(44.51) |  |
|  | Level2 | 320(9.69) | 195(7.87) |  |
|  | Level3 | 955(28.92) | 712(28.73) |  |
|  | Level4 | 339(10.27) | 468(18.89) |  |
| Susceptibility | |  |  | ＜0.001 |
|  | Level1 | 934(28.29) | 516(20.82) |  |
|  | Level2 | 1211(36.67) | 860(34.71) |  |
|  | Level3 | 616(18.66) | 397(16.02) |  |
|  | Level4 | 541(16.38) | 705(28.45) |  |
| Benefits | |  |  | ＜0.001 |
|  | Level1 | 1221(36.98) | 687(27.72) |  |
|  | Level2 | 1652(50.03) | 12.17(49.11) |  |
|  | Level3 | 429(12.99) | 574(23.16) |  |
| Barriers | |  |  | ＜0.001 |
|  | Level1 | 1193(36.13) | 1243(50.16) |  |
|  | Level2 | 321(9.72) | 209(8.43) |  |
|  | Level3 | 1261(38.19) | 737(29.74) |  |
|  | Level4 | 527(15.96) | 289(11.66) |  |
| Self-efficacy | |  |  | ＜0.001 |
|  | Level1 | 1202(36.40) | 712(28.73) |  |
|  | Level2 | 1627(49.27) | 1131(45.64) |  |
|  | Level3 | 473(14.32) | 635(25.63) |  |
| Trust in doctors | |  |  | ＜0.001 |
|  | Level1 | 991(30.01) | 573(23.12) |  |
|  | Level2 | 1182(35.80) | 713(28.77) |  |
|  | Level3 | 547(16.57) | 442(17.84) |  |
|  | Level4 | 582(17.63) | 750(30.27) |  |
| Trust in developers | |  |  | ＜0.001 |
|  | Level1 | 1320(39.98) | 745(30.06) |  |
|  | Level2 | 665(20.14) | 347(14.00) |  |
|  | Level3 | 826(25.02) | 686(27.68) |  |
|  | Level4 | 491(14.87) | 700(28.25) |  |

**Table S4. Balance test of PSM for urban and rural samples**

| Variables | Pre-PSM | | | Post-PSM | | |
| --- | --- | --- | --- | --- | --- | --- |
|  | Urban(M) | Rural(M) | *P* | Urban(M) | Rural(M) | *P* |
| Age | 3.015 | 3.296 | 0.002 | 3.216 | 3.191 | 0.555 |
| Sex | 1.549 | 1.505 | 0.001 | 1.522 | 1.522 | 0.974 |
| Religion | 1.026 | 1.072 | ＜0.001 | 1.042 | 1.038 | 0.516 |
| Educational level | 2.085 | 1.629 | ＜0.001 | 1.734 | 1.758 | 0.361 |
| Career | 1.047 | 1.041 | 0.038 | 1.044 | 1.046 | 0.817 |
| Self-report health condition | 80.384 | 81.421 | 0.005 | 81.161 | 80.932 | 0.655 |
| COVID-19 infection | 1.421 | 1.495 | 0.001 | 1.459 | 1.451 | 0.653 |
| Vaccine knowledge | 2.320 | 2.293 | ＜0.001 | 2.296 | 2.302 | 0.878 |
| Susceptibility | 2.231 | 2.521 | ＜0.001 | 2.366 | 2.316 | 0.150 |
| Barriers | 2.340 | 2.029 | ＜0.001 | 2.128 | 2.188 | 0.096 |
| Trust in doctors | 2.218 | 2.552 | ＜0.001 | 2.406 | 2.361 | 0.188 |
| Trust in developers | 2.148 | 2.541 | 0.019 | 2.386 | 2.336 | 0.165 |
| Vaccine accessibility | 1.729 | 1.808 | 0.017 | 1.734 | 1.739 | 0.829 |

NOTE：

M, mean.

PSM, Propensity Score Matching.

COVID-19, Coronavirus disease 2019.

**Table S5. The prevalence rates of COVID-19 vaccination in Chinese urban and rural settings post-PSM**

| Regions | Completed (95%CI) | Incompleted (95%CI) | *P* |
| --- | --- | --- | --- |
| Urban | 11.15(9.75-12.54) | 88.85(87.46-90.25) | ＜0.001 |
| Rural | 14.57(13.01-16.14) | 85.43(83.86-86.99) |  |

**Fig S1. Common support domain of PSM for urban and rural groups.**

**Table S6. Summary of research on prevention and control of COVID-19.**

| SN | Tiltle | Authors | Period | Region | Results |
| --- | --- | --- | --- | --- | --- |
| 1 | Rural-Urban Differences in Vaccination and Hesitancy Rates and Trust: US COVID-19 Trends and Impact Survey on a Social Media Platform, May 2021-April 2022 | Soorapanth S, Cheung R, Zhang X, Mokdad AH, Mensah GA. | May 2021 -April 2022 | The United States | 1. Two thirds of states showed statistically significant differences in monthly vaccination rates between rural and urban regions, with rural regions having a lower vaccination rate at all times.  2. Doctors and health professionals received the highest level of trust.  3. Friends and family were also among the most trusted sources in rural areas where the vaccination uptake was low. |
| 2 | A machine learning algorithm to analyse the effects of vaccination on COVID-19 mortality | Magazzino C, Mele M, Coccia M. | - | 192 countries | 1.At a certain point, the fatality rate collapses with increasing doses administered. |
| 3 | Rural-Urban Disparities in Hospital Admissions and Mortality Among Patients with COVID-19: Evidence from South Carolina from 2021 to 2022 | Giannouchos TV, Li Z, Hung P, Li X, Olatosi B. | January 2021 - January 2022 | South Carolina  (America) | 1. About 42% of all encounters resulted in an inpatient hospital admission, while hospital-level mortality was 6.3%.  2. Rural residents accounted for 31.0% of all encounters for COVID-19.  3. Rural residents had higher odds of overall hospital mortality, both as inpatients and as outpatients. |
| 4 | Sources, diffusion and prediction in COVID-19 pandemic: lessons learned to face next health emergency | Coccia M | - | - | 1. Surveillance of wildlife to avoid spillover effects with emergence and diffusion of new viral agents in humans.  2. Biosafety lab risk assessment and protocols to reduce accidents for the emergence and diffusion of new viral agents.  3. High air and environmental pollution, and (un)sustainable environment can support pandemic emergence and rapid spread.  4. Strengthening the early warning system with effective contract tracing system.  5. Effective public governance improves prevention and preparedness to face pandemic threats. |
| 5 | Examining Rural and Urban Sentiment Difference in COVID-19-Related Topics on Twitter: Word Embedding-Based Retrospective Study | Liu Y, Yin Z, Ni C, Yan C, Wan Z, Malin B. | May 2020 - January 2022 | the United States | 1. Rural users expressed stronger negative sentiments than urban users about COVID-19 prevention strategies and vaccination.  2. There was a clear political divide in the perception of politicians by urban and rural users; these users communicated stronger negative sentiments about Republican and Democratic politicians, respectively.  3.Regarding misinformation and conspiracy theories, urban users exhibited stronger negative sentiments about the "covidiots" and "China virus" topics, while rural users exhibited stronger negative sentiments about the "Dr. Fauci" and "pandemic" topics. |
| 6 | Effects of strict containment policies on COVID-19 pandemic crisis: lessons to cope with next pandemic impacts | Coccia M. | - | 31 countries | 1. Countries with a low intensity of strictness have average confirmed cases and fatality rates related to COVID-19 lower than countries with high strictness in containment policies.  2.High levels of strictness in public policy (and a high share of administering new vaccines) seem to have low effectiveness to stop pandemics similar to COVID-19 driven by mutant viral agents. |
| 7 | Influences, Barriers, and Facilitators to COVID-19 Vaccination: Cross-sectional Survey on Vaccine Hesitancy in 2 Rural States | Nguyen E, Wright M, Holmes J, et al. | June 2021 and July 2021 | Alaska or Idaho | 1. There were data from 736 usable surveys with 40 respondents who did not intend to be vaccinated, 27 unsure of their intentions, 8 who intended to be fully vaccinated with no doses received, and 661 fully vaccinated or who intended to be vaccinated with 1 dose received.  2. There were significant differences in characteristics and influences between those who were COVID-19 vaccine-hesitant and those who had been vaccinated. |
| 8 | COVID-19 Vaccination: Willingness and practice in Bangladesh | Faruk A, Quddus IA. | late January -early September 2021 | Bangladesh | 1. Willingness was not an issue in Bangladesh, but the weak link was getting individuals to register.  2. Confidence in public service delivery influenced favourable responses to mass immunization efforts. |
| 9 | Optimal levels of vaccination to reduce COVID-19 infected individuals and deaths: A global analysis | Coccia M. | March to May 2021 | 192 countries | 1. The average level of administering about 80 doses of vaccines per 100 inhabitants between countries can sustain a reduction of confirmed cases and number of deaths.  2. An intensive vaccination campaign in the initial phase of pandemic wave leads to a lower optimal level of doses administered per 100 inhabitants (roughly 47 doses of vaccines administered) for reducing infected individuals; however, the growth of pandemic wave (in May, 2021) moves up the optimal level of vaccines to about 90 doses for reducing the numbers of COVID-19 related infected individuals. |
| 10 | Global analysis of timely COVID-19 vaccinations: improving governance to reinforce response policies for pandemic crises | Benati I, Coccia M. | March 2021 | 112 countries | 1. Doses of vaccines administered × 100 inhabitants have a high positive association with the General Index of Governance.  2. An increase in the General Index of Governance improves the expected administration of doses of COVID-19 vaccines. |
| 11 | Factors influencing hesitancy towards adult and child COVID-19 vaccines in rural and urban West Africa: a cross-sectional study | Faye SLB, Krumkamp R, Doumbia S, et al. | 5 May - 5 June 2021 | Burkina Faso, Guinea, Mali, Senegal and Sierra Leone | 1. In West Africa at the time only 53% of all study participants reported to be aware of COVID-19 vaccines, and television (60%, n=1345), radio (56%; n=1258), social media (34%; n=764) and family/friends/neighbours (28%; n=634) being the most important sources of information about COVID-19 vaccines.  2. Perceived effectiveness and safety of COVID-19 vaccines increased the willingness to get vaccinated. |
| 12 | Improving preparedness for next pandemics: Max level of COVID-19 vaccinations without social impositions to design effective health policy and avoid flawed democracies | Coccia M. | - | 150 countries | 1.Vaccinations increase with the income per capita, achieving the maximum share of about 70% of total population, without coercion. |
| 13 | Perception towards vaccine effectiveness in controlling COVID-19 spread in rural and urban communities: A global survey | Marzo RR, Shrestha R, Sapkota B, et al. | April to August 2021 | different countries | 1. 64% of participants agreed that the vaccine effectively controlled viral spread, and 23% agreed that there was no need for vaccination if others were vaccinated.  2. Males had 14% higher odds of believing that there was no need for vaccination.  3. Less social media users had 39% higher odds of developing the belief that there is no need for vaccination than all other people vaccinated. |
| 14 | Rural-Urban Differences in the Associations Between Aging and Disability Services and COVID-19 Vaccination Rates Among Older Adults. | Sun Y, Rhubart DC. | March 1, 2022 | the United States | 1.Net of compositional characteristics, county-level density of aging and disability services is associated with higher older adult vaccination rates. |
| 15 | COVID-19 Vaccination is not a Sufficient Public Policy to face Crisis Management of next Pandemic Threats | Coccia M. | at the 11 January 2022 | 151 countries | 1.The increasing share of people vaccinated against COVID-19 seems to be a necessary but not sufficient health policy to reduce mortality of COVID-19. |
